# Thermal-driven H-bond reconfiguration for bioinspired high-strength anisotropic supramolecular hydrogels

**DOI:** 10.1039/d5mh02364e

**Published:** 2026-02-13

**Authors:** Jiayu Wu, Pan Jiang, XingXing Yang, Changcheng Bai, Ziyue Miao, Yixian Wang, Hang Zhang, Xiaolong Wang

**Affiliations:** a State Key Laboratory of Solid Lubrication, Lanzhou Institute of Chemical Physics, Chinese Academy of Sciences Lanzhou 730000 China wangxl@licp.cas.cn; b Department of Bioproducts and Biosystems, Aalto University Espoo 02150 Finland hang.zhang@aalto.fi; c Université Paris Cité, CNRS, Institut Jacques Monod F-75013 Paris France; d School of Chemistry and Chemical Engineering/State Key Laboratory Incubation Base for Green Processing of Chemical Engineering, Shihezi University Shihezi 832003 China; e Department of Applied Physics, Aalto University Espoo 02150 Finland

## Abstract

Natural anisotropic tissues, such as tendons and cartilages, achieve remarkable mechanical properties and various biofunctions through oriented hierarchical structures. Inspired from organisms, we develop a synergistic molecular and structural engineering technique based on the thermodynamically reversible reconfiguration of hydrogen bonds to achieve high-performance anisotropic supramolecular hydrogels by quenching pre-stretched polymer networks. Multiple inherent hydrogen bonds gradually dissociate under high-temperature processing to allow the good alignment of the polymer chains by uniaxial pre-stretching. The oriented polymer chains are then fixed on-site by rapid quenching-mediated hydrogen bond reconstruction at low temperatures (*e.g.*, ice bath). This process merely relies on the inherent hydrogen bonds of polymer chains instead of traditional salting-out, metal ionic coordination and solvent effects. The optimal anisotropic hydrogel shows a tensile strength of 19.4 ± 0.7 MPa and toughness of 53.8 ± 5.2 MJ m^−3^ along the pre-stretching direction, which are 2.6- and 1.7-times higher than that of the unquenched isotropic hydrogels, respectively. This general strategy is applicable to different strong hydrogen bonding supramolecular hydrogel systems. Furthermore, we fabricate anisotropic hydrogel fibers as damping materials. This general approach for the preparation of anisotropic supramolecular hydrogels shows great potential for various engineering applications, such as in the fabrication protective and cushioning materials, flexible optoelectronics, and mechano-functional scaffolds.

## Abstract 2

Inspired by organisms, we develop a hydrogen bond reconfiguration strategy to build supramolecular hydrogels *via* thermally reversible alignment. Quenching pre-stretched networks yields anisotropic hydrogels with outstanding mechanical performance.

## Graphical abstract

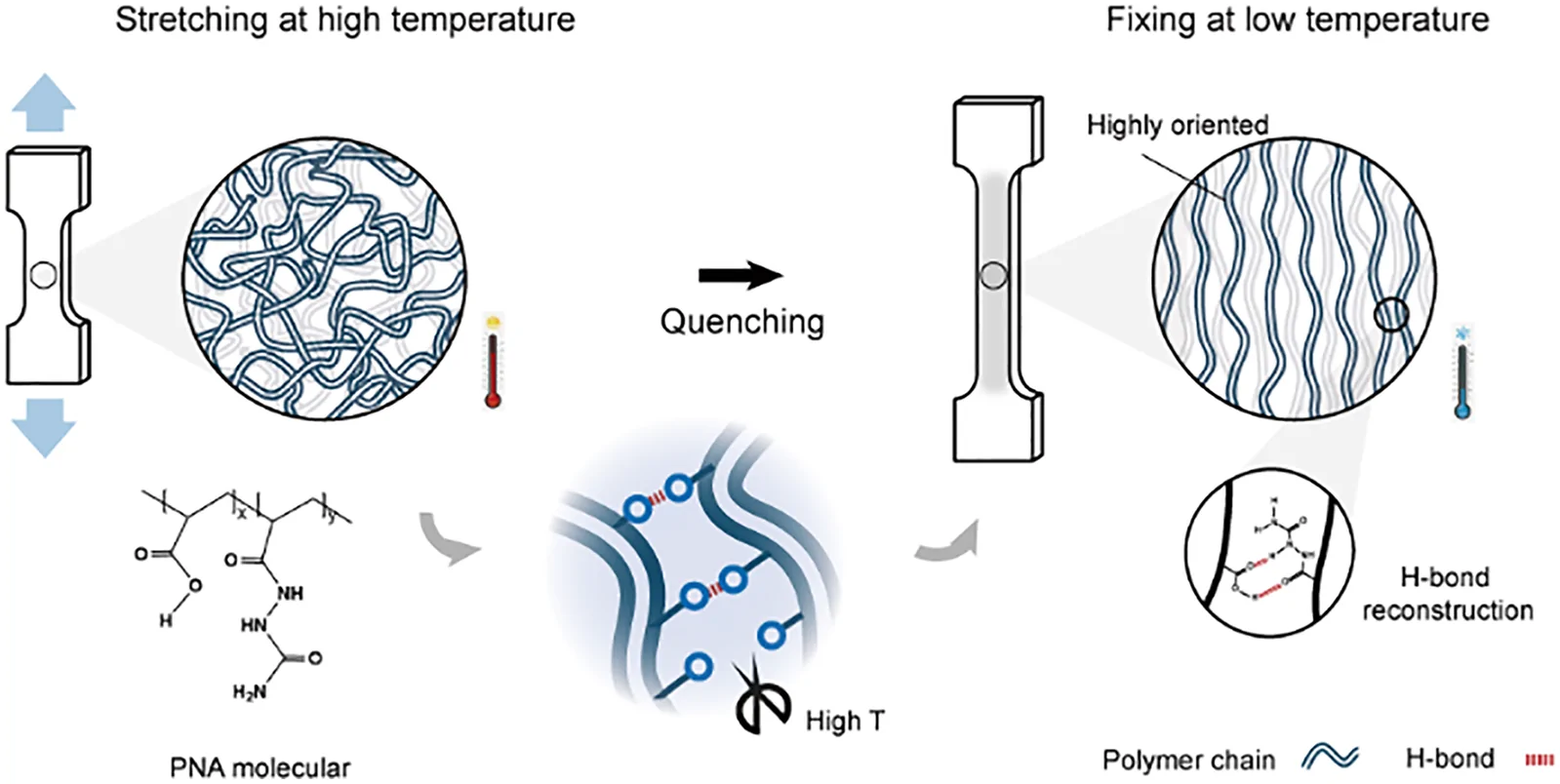

**New concepts**

In this study, we developed a rapid cooling strategy to control the reversible dissociation and reconstruction of hydrogen bonds in a supramolecular hydrogel. This strategy overcomes the classic challenge of fixing oriented polymer chains for high mechanical performance. By thermally dissociating and rapidly reforming inherent hydrogen bonds *via* quenching, we lock aligned structures without external additives. The obtained anisotropic hydrogels achieve a tensile strength of 19.4 ± 0.7 MPa and toughness of 53.8 ± 5.2 MJ m^−3^, significantly outperforming the isotropic hydrogels. We further demonstrate its versatility by fabricating strong, impact-resistant fibers and meshes, highlighting its potential for protective gear and flexible electronics.

## 1. Introduction

Natural anisotropic tissues, represented by load-bearing biological systems like tendons and articular cartilages, exhibit exceptional mechanical strength and fatigue resistance owing to the coordinated interactions between collagen fibers and soft proteoglycans, which form microstructures with multilevel orientations.^1,2^ To mimic these aligned architectures in biological systems, tremendous efforts have been devoted to creating anisotropic structures in hydrogels through freeze-casting,^3–9^ pre-stretching,^10,11^ micro-needle extrusion,^12,13^ compositing,^14^ and self-assembly,^15^ among others.^16–20^ The resultant high-performance anisotropic soft materials with unique aligned structures exhibit outstanding mechanical properties, asymmetric responses to external stimuli, directional conductivity and bio-mimic the biofunctions in comparison with isotropic ones. Therefore, this type of bioinspired soft materials has gained increasing popularity in various fields, such as soft actuators,^21,22^ artificial muscles,^23,24^ flexible electronics,^25^ and regenerative medicine.^26^

Structural engineering strategies based on directional ice templates,^9^ uniaxial stretching,^27^ repeated mechanical training,^28^ and shear flow induction^22^ have been utilized to directly achieve highly oriented structures in hydrogel matrices. However, a difficulty is maintaining the temporary orientation of the polymer chains when removing the external forces. It is often necessary to introduce molecular engineering strategies, including the salting-out effect, drying-induced polymer aggregation,^27,29^ metal ionic coordination,^27,30–33^ solvent exchange,^34^ annealing^4,6,35^ and physical cross-linking,^36,37^ to fix the aligned polymer structures *via* dynamic interactions at the molecular level. While such strategies can effectively “lock” the aligned polymer networks in the hydrogel, they often introduce trade-offs. For instance, natural drying may cause microcracks that weaken the mechanical integrity, while strategies like salting-out and metal-ion coordination can compromise the biocompatibility or network uniformity.^27,32^ In physically cross-linked polyvinyl alcohol (PVA) hydrogel systems, even though repeated mechanical training can enhance the molecular chain orientation and crystallinity, the inherent weak hydrogen bonding interactions remain insufficient to achieve high mechanical strength and long-term stability towards advanced engineering applications.^38–40^ This restricts the advanced design and engineering applications of biomimetic anisotropic hydrogels. Therefore, achieving a biomimetic balance in hydrogels, that is, creating materials that are highly orientable like biological tissues during their formation while being robustly stable like mature tissues solely through inherent molecular interactions remains a significant challenge.

Supramolecular hydrogels with designable dynamic interactions have a physically entangled network that is more susceptible to chain orientation under pre-stretching due to the absence of crosslinking junctions, thus promoting a higher degree of freedom for structural movement and alignment.^41^ Significant challenges remain in the development of high-performance supramolecular hydrogels with well-structured slip networks and effective fixed orientations, especially in exploring a balance among the degree of molecular migration, mechanical stability, and processing. To address this challenge, we employed a supramolecular hydrogel with strong multiple hydrogen bonding networks and developed a thermomechanical quenching strategy to control hydrogen bond dissociation and reconstruction. During high-temperature pre-stretching deformation, partial disruption of hydrogen bonds combined with the absence of covalent crosslinking in the supramolecular hydrogel matrix allows the polymer chains to slide more freely, thereby minimizing damage to the network structure and improving their alignment. Subsequently, rapid cooling of the heated pre-stretched hydrogels to 0 °C leads to the on-site reconstruction of dense multiple hydrogen bonds, which can effectively “lock” the oriented polymer chains. This process enables high chain mobility during deformation while achieving robust structural immobilization afterward using only intrinsic supramolecular interactions. In this way, quenching can solve the long-standing contradiction between high sliding features and stable immobilization. Most importantly, the optimal anisotropic hydrogels show high mechanical properties that merely rely on their inherent multiple hydrogen bonding interactions rather than externally introduced dynamic interactions. In additions, after quenching under large pre-stretching conditions, the supramolecular hydrogels exhibit a typical “J-shaped” stress–strain curve, leading to a high S/E (strength to modulus) ratio, similar to that of biological tissues. This thermomechanical programming approach presents a new quenching strategy for the fabrication of anisotropic supramolecular hydrogels, which has great potential in fields ranging from load-bearing biomedical devices to soft robotics.^22,42–44^

## 2. Results and discussion

### 2.1. Quenching supramolecular hydrogel

Based on the metallurgically inspired quenching strategy, we developed a pre-stretching protocol using a poly(*N*-acryloylsemicarbazide-*co*-acrylic acid) (PNA) hydrogel system that exhibits strong temperature-sensitive multiple hydrogen bond interactions.^45^ The copolymerization of *N*-acryloylsemicarbazide (NASC)^46,47^ and acrylic acid (AA) was initiated by lithium phenyl-2,4,6-trimethylbenzoylphosphinate (LAP) in a mixed dimethyl sulfoxide (DMSO)/deionized (DI) water solvent system (Fig. S1–S3). After solvent exchange in water to remove residual DMSO, the reconstruction of hydrogen bonds within the hydrogel matrix takes place, resulting in dense hydrogen-bonded networks of the PNA hydrogel. To identify the optimal hydrogel composition with high toughness and strength suitable for the subsequent quenching process, we systematically evaluated the mechanical properties of the PNA hydrogel across a range of monomer concentrations (20–40 wt%, designated as PNA_20_ to PNA_40_; Fig. S4–S6). Among these formulations, the PNA_25_ hydrogel with 25 wt% monomer concentration demonstrates an optimal balance between mechanical robustness and deformability, exhibiting a high tensile strength (7.5 ± 0.8 MPa) while maintaining exceptional elongation at break (981.4% ± 61.8%) and toughness (32.6 ± 5.4 MJ m^−3^) (Fig. S7). The high mechanical properties of the PNA_25_ hydrogel make it an ideal candidate for constructing anisotropic structures.

**Fig. 1:**
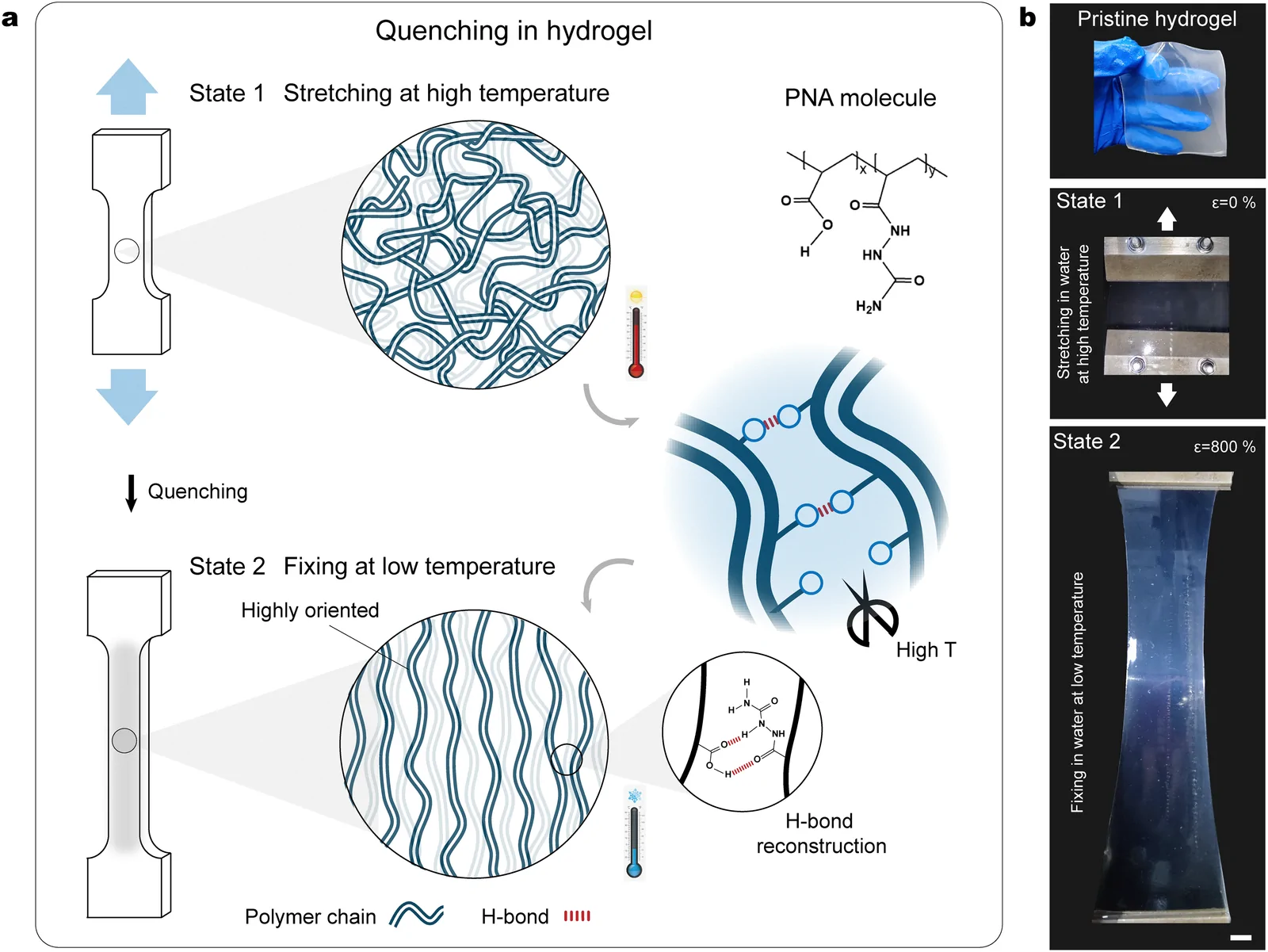
Schematic of the as-prepared anisotropic supramolecular hydrogels. (a) Mechanism of the process and (b) corresponding photos of hydrogel quenching after high-temperature stretching. The scale bar is 2 cm.

The fabrication of the anisotropic supramolecular hydrogels involved two key phases, including (1) hydrogen bond dissociation and (2) structural locking. Specifically, the PNA hydrogel was stretched in water at different temperatures (50–90 °C). In this case, the thermal energy disrupted the hydrogen bonds, allowing the polymer chains to align along the pre-stretching direction. Subsequently, the immediate quenching process in water at 0 °C allows the hydrogen bonds to rapidly re-form on site, effectively freezing the aligned hydrogel network (Fig. 1a). Importantly, this crosslinker-free supramolecular hydrogel system preserves its network integrity during the large pre-stretching process, preventing excessive structural damage. As a result, the final oriented supramolecular hydrogel exhibits a well-aligned polymer network, leading to improving mechanical properties while maintaining its structural integrity (Fig. 1b).

### 2.2. Anisotropic structure of quenched supramolecular hydrogel

**Fig. 2:**
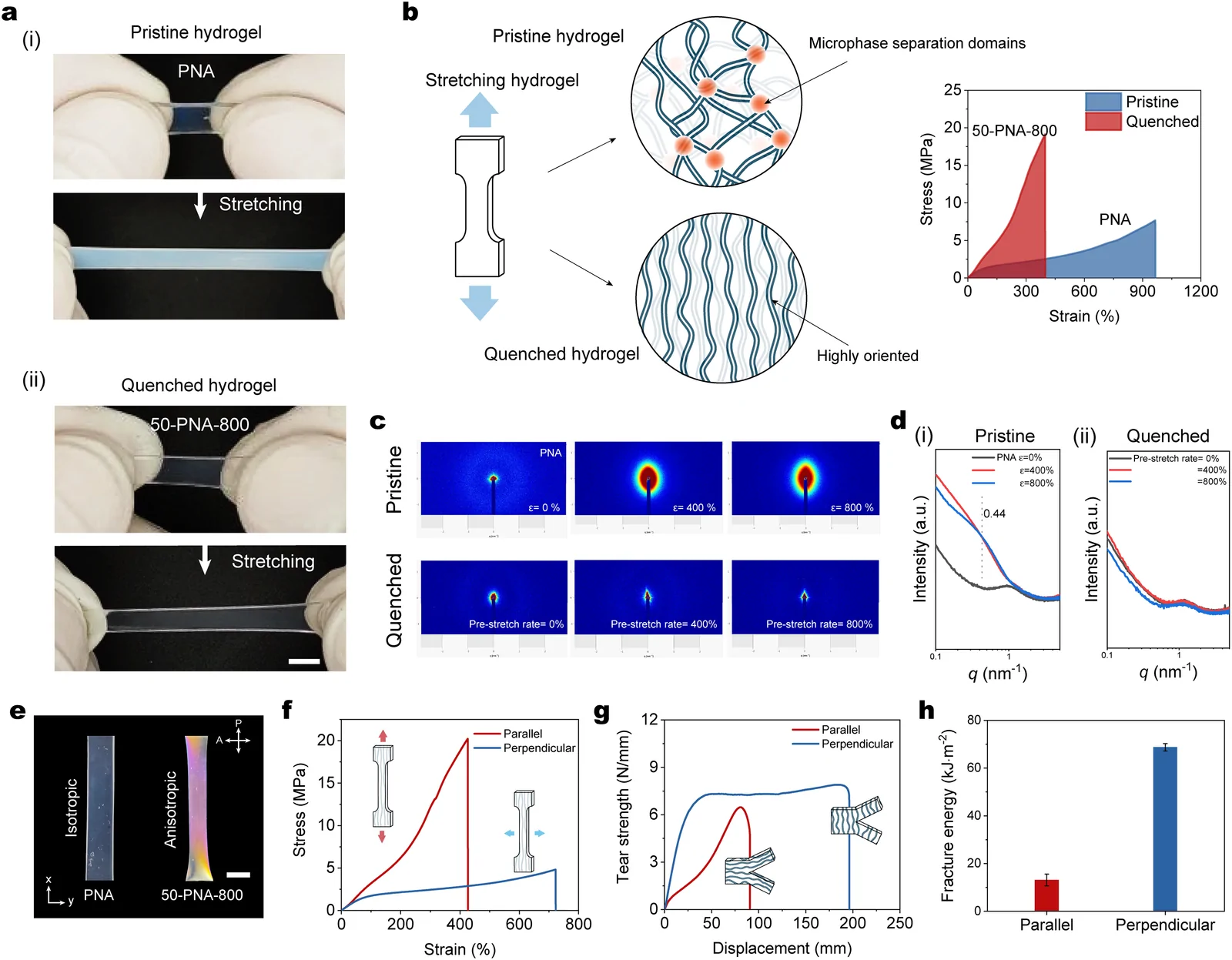
Anisotropic structures of hydrogels. (a) Digital photos of stretched hydrogels: (i) pristine and (ii) quenched. The scale bar is 5 mm. (b) Schematic of the network inside the pristine and quenched hydrogels. The inset on the right is the corresponding tensile stress–strain curve. (c) 1D SAXS intensity curves and (d) 2D map of the (i) pristine and (ii) quenched hydrogels at the same pre-stretch strain. (e) Polarized photographs of the pristine and quenched hydrogels. A and P indicate the directions of the analyser and polarizer, respectively. The scale bar is 5 mm. (f) Tensile stress–strain curves, (g) tear strength-displacement curves and (h) fracture energy of quenched hydrogels in parallel and perpendicular directions. Error bars represent the standard deviation (*n* = 3). The data are presented as mean value ± SD.

To elucidate the structural evolution of the polymer networks in our quenched supramolecular hydrogel system, we conducted a detailed investigation of the structural changes in the polymer chains before and after orientation. According to previous studies, the pristine hydrogels (PNA) exhibit stress whitening behavior during large deformation, which is attributed to the cleavage and remodeling of disordered hydrogen bonds, followed by the formation of microphase separation domains. This structural reorganization mechanism significantly enhances the tearing resistance through energy dissipation.^45^ The quenched supramolecular hydrogels are labeled as X-PNA-Y, where X represents the pre-stretch temperature and Y represents the pre-stretch strain. Notably, the 50-PNA-800 hydrogel (pre-stretched 800% at 50 °C followed by quenching at 0 °C) no longer exhibits significant microphase separation when stretched along the pre-aligned direction. The reason is that the polymer chains are already aligned along the stretching direction, resulting in a more uniform distribution of hydrogen bonds. Thus, the breaking and recombination of hydrogen bonds no longer drive the formation of distinct microphase separation domains in the isotropic PNA hydrogel (Fig. 2a and Video S1). Consequently, the quenching process inhibits microphase separation while significantly enhancing the stiffness and strength of the supramolecular hydrogels. As a result, the ultimate tensile strength of the quenched supramolecular hydrogels significantly increases to 19.4 ± 0.7 MPa, which is 2.6-times higher than that of the pristine isotropic hydrogels (Fig. 2b). Small-angle X-ray scattering (SAXS) analysis demonstrates the structural changes before and after quenching. The results indicate that as the pre-stretching strain gradually increases to 800% for the pristine hydrogels, a distinct peak is observed at a *q*-value of 0.44 nm^−1^, confirming the formation of phase-separated domains. The reason is that the linear polymer chains in the supramolecular polymer hydrogels undergo elastic deformation and plastic slip along the tensile direction during the stretching process. Accordingly, the microstructural evolution of the hydrogels rearranges from random coils to orderly arrangements, which subsequently drives domain assembly. In contrast, the quenched samples of the 50-PNA-400 and 50-PNA-800 hydrogels are unable to produce any new phase-separated domains. This is because the polymer chains are already oriented along the stretching axis, resulting in a more uniform distribution of hydrogen bonds. Consequently, the breaking and reformation of hydrogen bonds during stretching do not drive the formation of new distinct microphase domains. Domains are only intrinsically present at *q* = 1.13 nm^−1^ owing to the hydrogen-bonded nano-domains (Fig. 2c and d). The azimuth plot of the 2D SAXS pattern of the 50-PNA-800 hydrogel has a peak at the azimuth angle of 0°, corresponding to the orientation order parameter of 0.87, while the pristine PNA hydrogel does not have an obvious peak (Fig. S8). The polarized image in Fig. 2e shows that the quenched hydrogel has an obvious birefringence phenomenon, demonstrating the parallel orientation of the molecular chains to this plane. Additionally, scanning electron microscopy (SEM) characterization also confirms the evolution of the anisotropic architecture within the quenched supramolecular hydrogels at different pre-stretching strain. It can be found that the quenched supramolecular hydrogels demonstrate a clearly oriented structure, and meanwhile preserve an integrated dense network architecture without discernible structural imperfections (Fig. S9). The slight decrease in water content after quenching is responsible for the reorganization of the polymer chains into more ordered and densely packed structures. This structural rearrangement promotes the assembly of an enhanced multiple hydrogen bonding network (Fig. S10).

The anisotropic mechanical properties further confirm the anisotropy structure of the quenched supramolecular hydrogels. As shown in Fig. 2f, the tensile strength of the quenched supramolecular hydrogels parallel to the pre-stretch direction is approximately 4-times higher compared to the vertical direction. To further evaluate its stability under prolonged hydration, we conducted mechanical tests on the 50-PNA-800 hydrogel sample after 14 days of post-quenching equilibrium. The stress–strain curves reveal that there is no significant change in tensile strength (Fig. S11). This demonstrates that the oriented structure after quenching maintains good mechanical stability. The toughness in the parallel orientation reaches 33.3 ± 2.2 MJ m^−3^, which is about 1.5-times that in the vertical direction. Additionally, the tearing energy in the parallel direction is 13.1 ± 2.4 kJ m^−2^, while it reaches 68.7 ± 1.6 kJ m^−2^ in the vertical direction (Fig. 2g and h). The above-mentioned results support that the quenching strategy significantly enhances the mechanical properties along the oriented direction by fixing the highly anisotropic structure in the supramolecular hydrogel matrix.

### 2.3. Thermally reversible hydrogen bonds

**Fig. 3:**
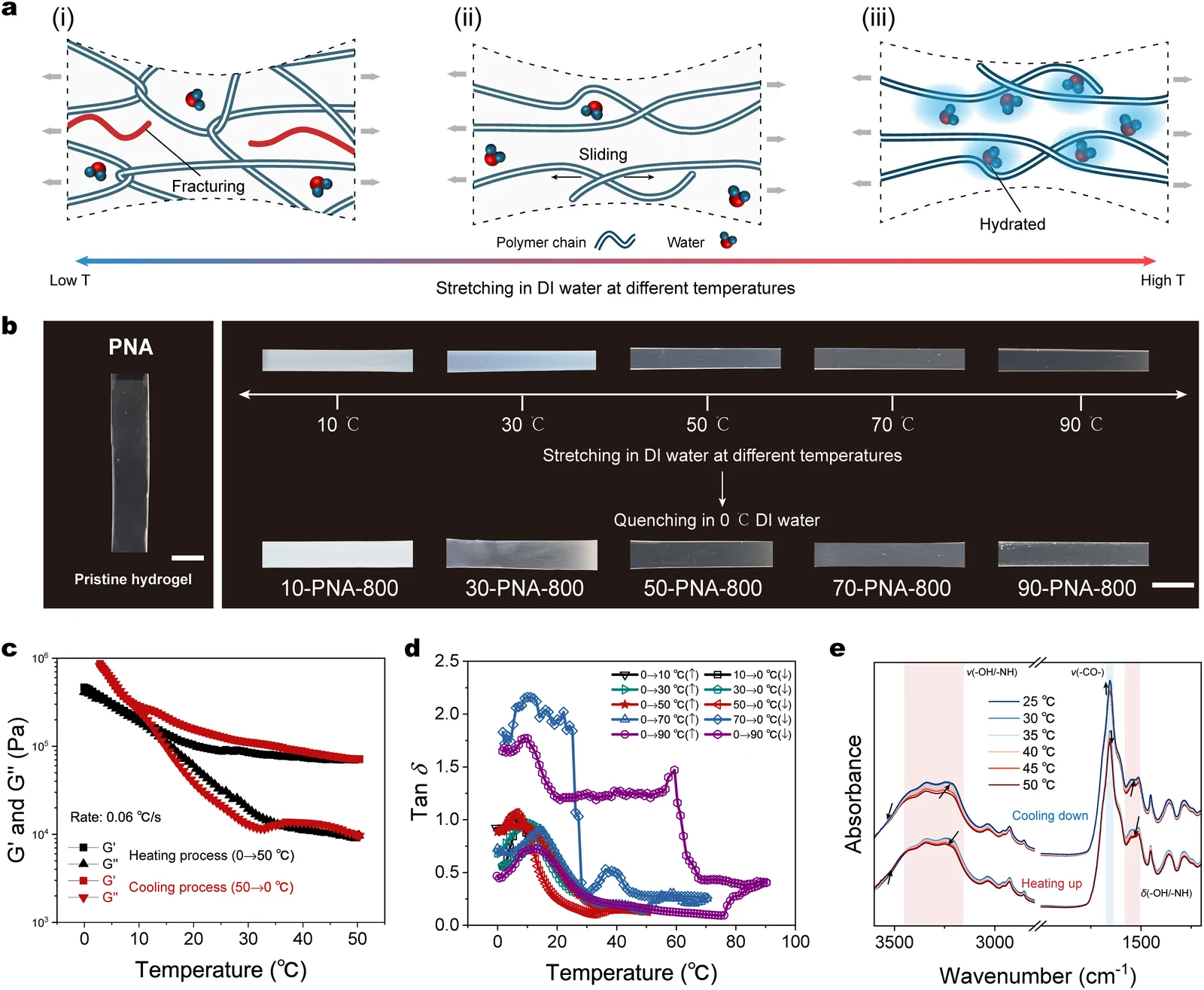
Effect of temperature on the pre-stretching hydrogel performance. (a) Schematic of polymer network reorganization during stretching at varying temperatures. (b) Digital photographs of hydrogels quenched at different temperatures. The scale bar is 5 mm. (c) Rheological test of the PNA hydrogel from 0 °C to 50 °C and from 50 °C to 0 °C and the corresponding (d) tan *δ* curves. (e) Temperature-dependent infrared spectra. The heating and cooling rate was set to 2 °C min^−1^, with data collected every 5 °C.

Physically crosslinked supramolecular hydrogel networks contain strong multiple hydrogen bonds that exhibit temperature-sensitive character. Thus, it is necessary to optimize the heating temperature for pre-stretching to achieve favorable weakening of the hydrogen bonding interactions, enabling effective chain rearrangement and alignment during uniaxial deformation (Fig. 3a). Herein, the supramolecular hydrogels were subjected to 800% pre-stretching deformation at different temperatures followed by rapid quenching in ice water at 0 °C for 2 min. They were labeled as 10-PNA-800, 30-PNA-800, 50-PNA-800, 70-PNA-800, and 90-PNA-800 based on their initial processing temperature. Accordingly, lower processing temperatures significantly restrict the polymer chain mobility by enhancing hydrogen bonds, leading to localized stress concentrations, and eventually chain fracture. This is exemplified by the 10-PNA-800 hydrogel, which exhibits internal network damage characterized by micro-void formation and distinct whitening phenomena. However, when the processing temperature is close to 90 °C, the hydration-induced hydrophilic groups originating from complete dissociation of hydrogen bonds are seriously hydrated, which inhibits the reconstitution of hydrogen bonds between the polymer chains after quenching, resulting in the swelling of the 90-PNA-800 hydrogel (Fig. 3b). The temperature-dependent evolution of the hydrogen bonds within the polymer chains can be further elucidated by temperature-dependent rheology and FTIR analyses. As shown in Fig. 3c and Fig. S12, with an increase in temperature, both the storage modulus (*G'*) and loss modulus (*G''*) of the pristine hydrogels gradually decrease. According to tan *δ*, hydrogen bond dissociation becomes particularly pronounced in the range of about 10–40 °C, while the range of about 40–50 °C exhibits a relatively stable phase (Fig. 3d). Upon cooling from 50 °C, the values of *G'* and *G''* are almost fully recovered, indicating reversible hydrogen bonding interactions. In contrast, when heated to 70 °C or 90 °C, the hydrogels fail to recover to their original modulus level. These results suggest that the upper temperature limit for effective reversible hydrogen bonding interactions is approximately 50 °C. This conclusion is further supported by the temperature-dependent FTIR analysis (Fig. 3e). The intensities of the hydrogen bond donor peaks ν –OH (3268 cm^−1^) and ν –NH (3440 cm^−1^, 3342 cm^−1^ and 3207 cm^−1^) as well as the acceptor peak ν –COOH (1661 cm^−1^) decrease upon heating due to hydrogen bond disruption while increase upon cooling owing to the reformation of the hydrogen bonds. These results confirm that the heating temperature of 50 °C is a desirable range to make hydrogen bonds exhibit good reversibility and dynamic reconstruction capability.

### 2.4. Mechanical behavior of quenching supramolecular hydrogels

**Fig. 4:**
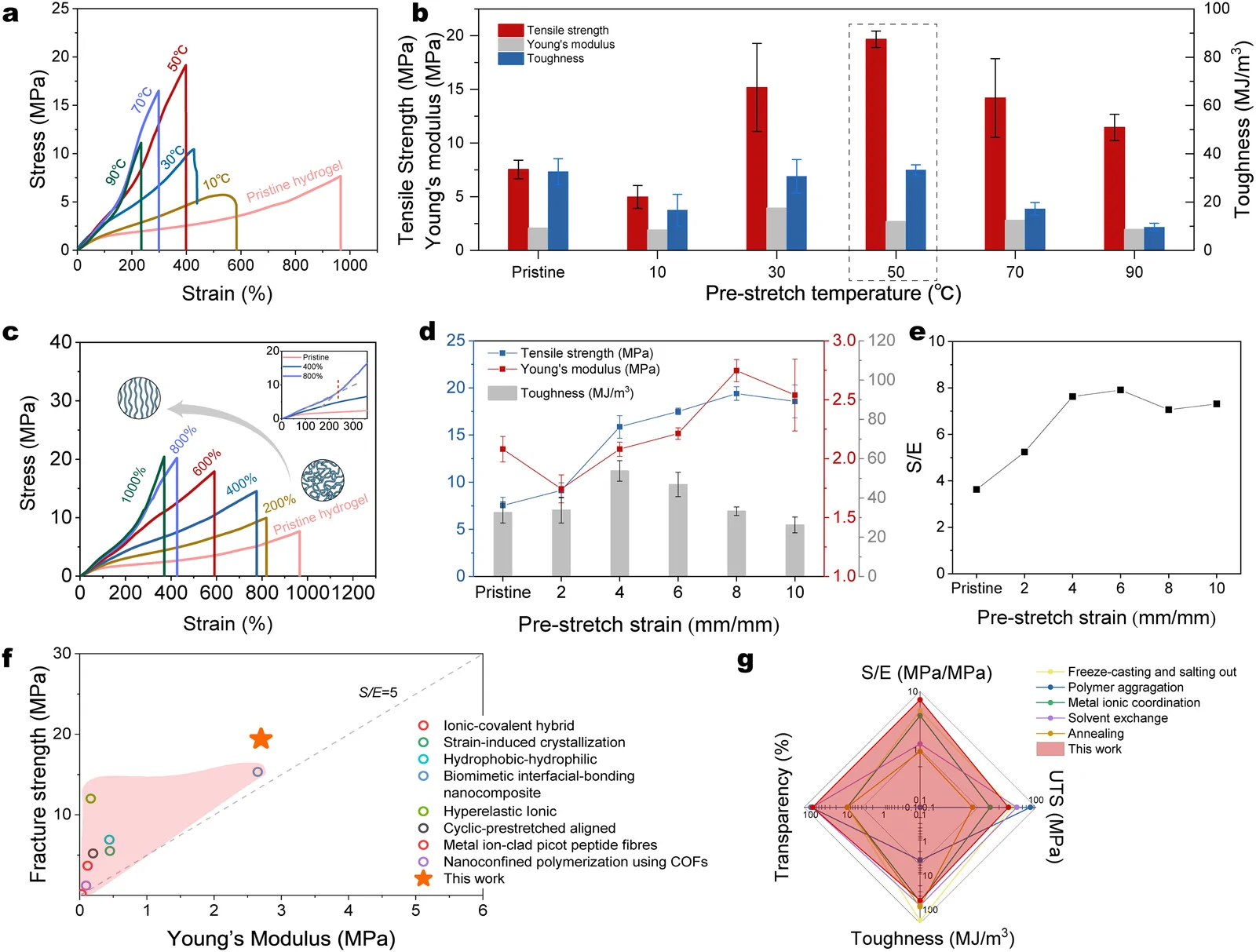
Influence of quenching parameters on the structural and mechanical performance of hydrogels. (a) Tensile stress–strain curves of the pristine hydrogel and quenched hydrogels after being stretched to 800% at different temperatures, along with the corresponding (b) tensile strength, Young's modulus and toughness. (c) Tensile stress–strain curves of hydrogels quenched after being stretched to different pre-stretch strain at 50 °C, along with the corresponding (d) tensile strength, Young's modulus, and toughness. Error bars represent the standard deviation (*n* = 3). The data are presented as mean value ± SD. (e) Strength-to-modulus ratio (S/E) of the pristine and quenched hydrogels. (f) Comparison chart in the Ashby plot of Young's modulus *versus* fracture strength of quenched hydrogel with other reported strong and tough hydrogels. Examples of hydrogels with an S/E ratio greater than 5 include ionic-covalent hybrid,^48^ strain-induced crystallization,^49^ hydrophobic–hydrophilic,^50^ biomimetic interfacial-bonding nanocomposite,^51^ hyperelastic ionic,^52^ cyclic-prestretched aligned,^39^ metal ion-clad picot peptide fibres,^53^ nanoconfined polymerization using COFs.^54^ (g) Radar map comparing UTS, toughness, S/E ratio, and transparency across various anisotropic hydrogel types, including freeze-casting and salting out,^9,55^ polymer aggregation,^56,57^ metal ionic coordination,^58^ solvent exchange and annealing.^4,6^

Building upon these insights into temperature-dependent hydrogen bonding behavior, we investigated how the heating temperature for pre-stretching impacts the mechanical performance of the quenched supramolecular hydrogels. As shown in Fig. 4a, the structural defects in 10-PNA-800 resulted in the tensile fracture strength dropping to 5.0 ± 1.1 MPa and the Young's modulus decreasing to 1.9 ± 0.1 MPa, which are both lower than that of the pristine PNA hydrogels. It should be noted that the abovementioned rheological data shows that the interchain hydrogen bonds are largely dissociated at temperatures exceeding ∼ 40 °C. Therefore, elevating the processing temperature to 50 °C substantially enhances the chain mobility of 50-PNA-800 through partial dissociation of the hydrogen bonds, which could improve the slippage capability of the polymer chains to facilitate smoother chain alignment during uniaxial stretching. Upon quenching, this oriented structure is “frozen”, forming a more ordered yet denser molecular network, thereby significantly improving both tensile strength and modulus. Specifically, the tensile strength and Young's modulus increase to 19.4 ± 0.7 MPa and 2.7 ± 0.1 MPa, respectively, with high toughness (33.3 ± 2.2 MJ m^−3^) along the pre-stretching direction (Fig. 4b). When the heating temperature exceeds 70 °C, hydration of the hydrophilic groups inhibits the reconstitution of intermolecular hydrogen bonds after quenching, resulting in degradation of mechanical properties. Observations of the lateral size variation before and after quenching further indicate that the shrinkage of the hydrogel slows down significantly when the pre-stretching temperature exceeds 70 °C (Fig. S13). Overall, the above-mentioned systematic parameter evaluation identifies that pre-stretching at 50 °C is the optimal temperature to maximumly balance the strength-modulus characteristics, as well as the stability of the oriented structure (Fig. S14). We also investigated the effects of quenching parameters on the kinetics of hydrogen bond reconstruction and mechanical performance. As shown in Fig. S12, a short quenching time of 10 s resulted in insufficient hydrogen bond reformation and a tensile strength of 12.7 ± 1.5 MPa. Increasing the quenching time to 30 s significantly enhanced the tensile strength to 17.2 ± 1.0 MPa, while further extension to 2 min yielded a value of 19.4 ± 0.7 MPa, indicating that effective hydrogen bond reconstruction was achieved within 30 s at 0 °C (Fig. S15). Meanwhile, medium temperature strongly influenced the stabilization of the oriented polymer chains. As the temperature decreased from 30 °C to 0 °C, the tensile strength showed a notable improvement, reflecting enhanced hydrogen bond interactions at lower temperatures (Fig. S16).

Subsequently, we studied the effect of pre-stretching strain on the mechanical properties of the supramolecular hydrogels treated at 50 °C. As shown in Fig. 4c and d, the tensile strength and Young's modulus of the supramolecular hydrogel exhibit a non-monotonic dependence on pre-stretching strain, reaching peak values at 800% strain followed by performance degradation, while the 50-PNA-400 hydrogel exhibited the maximum toughness (53.8 ± 5.2 MJ m^−3^) at 400% strain, representing a 1.6-times enhancement over the pristine hydrogel. Mechanistic analysis of the stress–strain curves reveals two critical transitions: (1) gradual disappearance of yield points beyond 400% pre-stretching and (2) emergence of negative curvature inflection points at 800% strain, indicating weakened plastic deformation capacity in the polymer networks. In particular, the strain-hardening behavior observed here results in a J-shaped stress–strain curve, which closely resembles those commonly found in biological tissues. As shown in Fig. 4e, the hydrogel exhibited a peak strength-to-modulus ratio (S/E = 7.8) at ∼600% strain, demonstrating concurrent optimization of mechanical compliance and tensile robustness through this critical deformation threshold. To compare our results with existing hydrogels, we summarized the tensile strength and Young's modulus of various strong and tough hydrogels in Fig. S17, and specifically highlighted those with an S/E ratio greater than 5 in Fig. 4f. Remarkably, the quenched supramolecular hydrogel exhibits outstanding tensile fracture strength at this level of strength-modulus ratio S/E, which indicates the comprehensive amplification in flexibility together with mechanical strength. Moreover, existing anisotropic hydrogels have yet to achieve a combination of high strength, flexibility and high transparency. Following our design strategy, the prepared hydrogel exhibits high transparency, with the optical transmittance of approximately 90% at 550 nm, and its overall performance surpasses that of most reported anisotropic hydrogels (Fig. 4g and Fig. S18).

### 2.5. Processability and applications

**Fig. 5:**
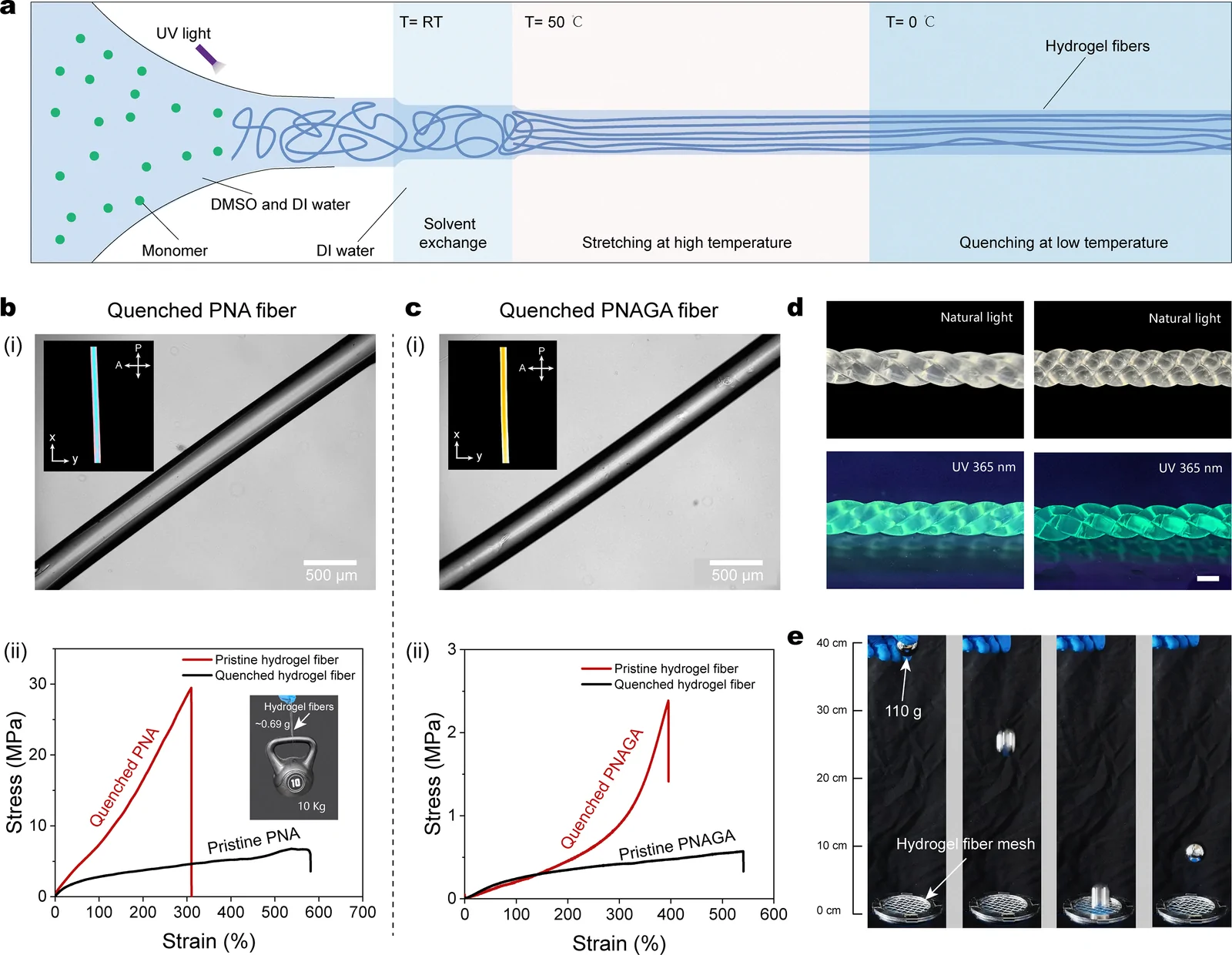
Preparation and applications of hydrogel fibers. (a) Schematic of the hydrogel fiber preparation process. (b) (i) Microscopic photographs of the quenched PNA hydrogel fibers. The inset shows the polarized photograph of the quenched PNA hydrogel fibers. A and P indicate the directions of the analyzer and polarizer, respectively. (ii) Tensile stress–strain curve of the pristine and 50-PNA-800 hydrogel fiber. The inset is lifting a dumbbell using the 50-PNA-800 hydrogel fibers. (c) (i) Microscopic photographs of quenched PNAGA hydrogel fibers. The inset shows the polarized photograph of the quenched PNAGA hydrogel fibers. A and P indicate the directions of the analyser and polarizer, respectively. (ii) Tensile stress–strain curve of the pristine and the quenched PNAGA hydrogel fiber. (d) Natural light and fluorescence-stained photographs of hydrogel fibers with different weaving patterns. The scale bar is 0.5 mm. (e) Drop-weight impact test of the hydrogel fiber mesh.

The quenched supramolecular hydrogels can be integrated into the extrusion process to fabricate hydrogel fibers that meet the needs of extreme mechanical applications in various engineering fields. As a demonstration, we designed and fabricated hydrogel fibers using an extruder followed by ultraviolet exposure to form hydrogel fiber precursors. After equilibration in water, the fibers underwent pre-stretching of 800%, and heating at 50 °C and quenching at 0 °C to complete all the processes (Fig. 5a and Fig. S19). The resulting 50-PNA-800 hydrogel fibers exhibit exceptional mechanical properties and structural integrity. Notably, compared to the sheet-shaped 50-PNA-800 hydrogels, the quenched cylindrical hydrogel fibers display a more uniform structure and enhanced mechanical strength, achieving a tensile strength of ∼29.5 MPa (Fig. 5b). The 50-PNA-800 hydrogel fiber, weighing only ∼0.69 g, can endure a heavy load of up to 10 kg without breaking. Besides, this strategy demonstrates excellent universality, which was successfully extended to another supramolecular hydrogel system with multiple strong hydrogen bonds, *i.e.*, poly(*N*-(2-amino-2-oxoethyl)acrylamide) (PNAGA). It can be found that the tensile strength of the PNAGA hydrogel after quenching is enhanced by 4.2-times compared to the pristine isotropic hydrogel (Fig. 5c). Beyond their mechanical robustness, the hydrogel fibers offer excellent flexibility, enabling complex weaving and twisting. Furthermore, by immersing them in fluorescent molecules, the fibers acquire fluorescence properties, making them promising candidates for applications such as visualized fluorescent surgical sutures^59,60^ (Fig. 5d). Additionally, the soft and flexible 50-PNA-800 hydrogel fibers can be woven into a mesh with outstanding impact resistance, capable of withstanding the impact of a 110 g steel ball without damage (Fig. 5e and Fig. S20 and Video S2). Overall, the proposed quenched hydrogel fibers hold significant potential for applications in biomedical engineering, protective and cushioning materials, flexible electronics, and smart devices.

## 3. Conclusions

In this study, we developed high-performance anisotropic supramolecular hydrogels *via* their quenching-mediated inherent hydrogen bonding reconfigurations. The temperature-sensitive hydrogen bonding networks allow the linear polymers to undergo effective slippage and orientation during high-temperature pre-stretching. Quenching, *i.e.* rapid cooling, promotes multiple hydrogen bonding reconstitution to freeze the oriented structure. Without the common need for external fixation cues, this approach significantly improves the tensile strength (2.6 times) and toughness (1.7 times) of the supramolecular hydrogels, especially along the orientation direction. Notably, by optimizing the heating temperature and pre-stretching strain, the resultant anisotropic supramolecular hydrogel not only exhibits superior mechanical properties but also transitions from its original plasticity to J-type strain hardening behavior with a high S/E ratio, which is very similar to biological tissues. This strategy is generalizable to hydrogen-bonded hydrogel networks and possesses excellent processability, as demonstrated by the potential paradigms of hydrogel fibers and woven meshes. The fibers and braided structures exhibit excellent flexibility and impact resistance, and offer promising applications in biomedical engineering, protective materials, and flexible electronics. Overall, this simple, efficient, and green quenching strategy provides a powerful method to design high-performance anisotropic supramolecular hydrogels, paving the way towards broad applications across diverse engineering fields.

## 4. Materials and methods

### 4.1. Materials

Acrylic acid (99.0%, TCI, Shanghai, China), acryloyl chloride (98%, Aladdin, Shanghai, China), semicarbazide hydrochloride (98%, Acmec, Shanghai, China), *N*-(2-amino-2-oxoethyl)acrylamide (99.98%, Bellen (Dalian) Chemistry Co., Ltd, China), diethyl ether (99.5%, Kelong Company, China), anhydrous potassium carbonate (K_2_CO_3_, 99%, Aladdin, Shanghai, China), and lithium phenyl-2,4,6-trimethylbenzoylphosphinate (LAP) were used in this study. Deionized (DI) water was prepared in the laboratory.

### 4.2. Preparation of the PNA hydrogel

The NASC and AA monomers were dissolved in a binary solvent system consisting of DMSO and deionized (DI) water with a mass ratio of 7 : 3 and a monomer mass ratio fixed at NASC : AA = 1 : 0.4. Lithium phenyl(2,4,6-trimethylbenzoyl)phosphinate (LAP, 0.5 wt% of the total monomer mass%) was added as a photoinitiator. After irradiating the precursor solution with UV light for 2 min to complete polymerization, the prepared hydrogel was immersed in deionized water for 7 days to remove the DMSO solvent, resulting in a balanced PNA hydrogel.^45^

### 4.3. Quenching process of the PNA hydrogel

The hydrogel samples were clamped in a custom-made stretching unit equipped with a servo-motor drive system and pre-stretched in deionized water at 10–90 °C. The strain ratio (1 to 10 times the initial length) was systematically programmed *via* a digital control panel. Immediately after the hydrogel reached the target pre-stretch strain, it was quenched in deionized water at 0 °C for 2 min to reconstruct the fixed-oriented structure using fast hydrogen bonding. Finally, the quenched hydrogel was transferred to deionized water at 25 °C for equilibration for 7 days to obtain the quenched hydrogel.

### 4.4. Preparation of the quenched hydrogel fibers

The total weight of 25 wt% of NASC and AA monomers was dissolved in a mixed solvent of DMSO and deionized water (mass ratio 7 : 3), where NASC : AA = 1 : 0.4, and LAP (0.5 wt% of the total mass of the monomer) was added as a photoinitiator. The hydrogel precursor solution was then extruded at a constant speed through a pressure-driven transparent PDMS tubing device while being irradiated with 405 nm light to initiate photopolymerization and crosslinking of the monomers. The as-prepared hydrogel fibers were then immersed in DI water for 7 days to completely remove the DMSO solvent. Subsequently, the hydrogel was stretched 8-fold at 50 °C using a home-made stretching machine, and then quickly put in 0 °C water for 2 min, and finally equilibrated in 25 °C water. The resultant anisotropic hydrogel fibers were immersed in a 0.1 M fluorescein sodium aqueous solution and incubated under light-protected conditions for 6 h to allow sufficient diffusion of the fluorescent molecules into the hydrogel network. The fibers were repeatedly rinsed with deionized water to remove superficially adsorbed dye.

### 4.5. Mechanical properties

Uniaxial tensile testing was performed using a universal testing machine (EZ-Test, Shimadzu, Japan) equipped with a 2000 N load cell. The spline was measured in a rectangular and fibrous hydrogel row. The tensile test was performed in water at 100 mm min^−1^, and the Young's modulus was determined by the slope of the stress–strain curve over a strain range of 1% to 10%, with each data point representing the average of at least three samples.

For the tear test, a hydrogel sample with a width (*w*) of 10 mm and length of 40 mm was prepared, and an initial notch of 20 mm was introduced in its center along the length using a cutter. During the test, one side of the sample was fixed to the base, while the other leg was clamped to the beam, which moved at a speed of 100 mm min^−1^ in water. After the test, the tear strength-displacement curve was recorded, and the tear energy of the sample was calculated using the following formula:1
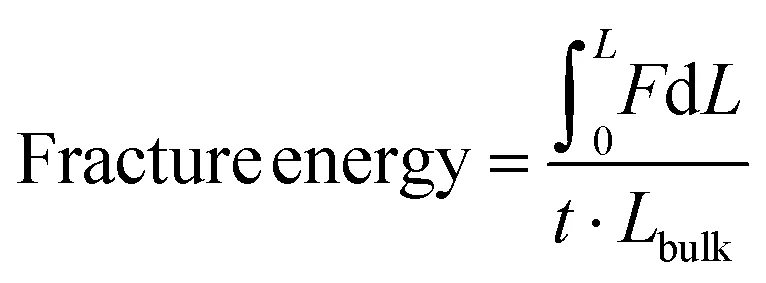
where *F* is the tearing force, *t* is the sample thickness, *L* is the displacement, and *L*_bulk_ is the projected crack length.

### 4.6. Water content measurement

The water content of the hydrogels was determined by measuring their weight before and after water removal. It was expressed as a percentage of the total weight of the hydrogel and calculated using the following equation:2
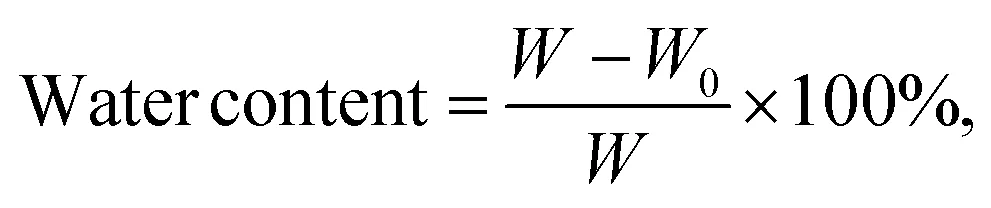
where *W* and *W*_0_ are the weights of the hydrogel before and after drying, respectively.

### 4.7. Temperature-dependent FTIR

The hydrogel film (diameter: 15 mm, thickness: 0.95 mm) was mounted on ZnSe sheets, and infrared transmission tests were conducted using a Thermo Fisher Scientific IS50/HPR20 infrared spectrometer. The heating rate was set to 2 °C min^−1^, with data collected every 5 °C from 25 °C to 50 °C. During cooling, data were collected every 5 °C from 50 °C to 25 °C, with a cooling rate of 2 °C min^−1^. As a result, a temperature-variable infrared spectrum was obtained.

### 4.8. Rheological properties

The hydrogel film (diameter: 25 mm, thickness: 1.2 mm) was placed on a variable temperature platform and tested using an Anton Paar Physica MCR 302 rheometer at a frequency of 1 Hz and strain of 1%. The temperature was increased from 0 °C to 10 °C, 30 °C, 50 °C, and 90 °C at a heating rate of 0.06 °C min^−1^, and then cooled back to 0 °C at the same cooling rate of 0.06 °C min^−1^.

### 4.9. Characterization instruments and methods

Fourier transform infrared (FT-IR) spectroscopy was recorded using a TFS-66 V/S spectrometer (Bruker) using the KBr-disk method. Nuclear magnetic resonance (NMR) spectroscopy was recorded using a Bruker Avance Neo 400WB. The transmittance test was conducted using a UV-Vis-NIR Agilent Cary 5000 instrument. The samples used for small angle X-ray scattering (SAXS) were examined by small angle X-ray scattering (Anton Paar SAXSpoint 2.0). The orientation index (*π*) was calculated from the intensity distribution profile along the azimuthal angle using the equation *π* = (180 − *H*)/180, where *H* is the half-width at half-maximum of the peak in the azimuthal plot obtained. The hydrogels under different strains were cold-dried at −40 °C and then tested. The microscopic morphology of the hydrogel was determined by scanning electron microscopy (SEM; Netherlands Phenom ProX), with the voltage of 10–30 keV. Specifically, the cross-section of the hydrogel sample was obtained by mechanical bending after quenching with liquid nitrogen.

## Author contributions

X. W. and P. J. co-designed and conceived the project. J. W. designed and performed all experiments. X. W. supervised the project. J. W., P. J., H. Z., and X. W. wrote the manuscript. J. W., P. J. and H. Z. analyzed the data. Z. M. performed rheology tests. X. Y. helped with temperature-dependent infrared spectroscopy. All authors contributed to the analysis and discussion of the results.

## Conflicts of interest

The authors declare no competing financial interest.

## Supplementary Material

MH-013-D5MH02364E-s001

MH-013-D5MH02364E-s002

MH-013-D5MH02364E-s003

## Data Availability

The authors declare that data supporting the findings of this study are available within the paper and its supplementary information (SI) files. The data is available from the authors on request. Source data are provided with this paper. Supplementary information is available. See DOI: https://doi.org/10.1039/d5mh02364e.
