# Supplementary material for: Thermal-driven H-bond reconfiguration for bioinspired high-strength anisotropic supramolecular hydrogels
Source: Mater Horiz. 2026 Feb 13;13(9):4430–40. doi: 10.1039/d5mh02364e (PMC13154373; doi:10.1039/d5mh02364e)
Supplement: MH-013-D5MH02364E-s001 [file MH-013-D5MH02364E-s001.pdf]

Supplementary Information (SI) for Materials Horizons.
This journal is © The Royal Society of Chemistry 2026

Supporting Information

Thermal-driven H-bond reconfiguration for bioinspired high-strength anisotropic supramolecular hydrogels

Jiayu Wu, [ab†] Pan Jiang, [c†] XingXing Yang,[d] Changcheng Bai,[a] Ziyue Miao,[e] Yixian Wang,[d] Hang Zhang, [b*] Xiaolong Wang [a*]

a. State Key Laboratory of Solid Lubrication, Lanzhou Institute of Chemical Physics, Chinese Academy of Sciences, Lanzhou 730000, China.

b. Department of Bioproducts and Biosystems, Aalto University, Espoo 02150, Finland.

c. Université Paris Cité, CNRS, Institut Jacques Monod, F-75013 Paris, France.

d. School of Chemistry and Chemical Engineering/State Key Laboratory Incubation Base for Green Processing of Chemical Engineering, Shihezi University, Shihezi 832003, China.

e. Department of Applied Physics, Aalto University, Espoo 02150, Finland.

† These authors contributed equally.

Supporting Methods

Synthesis of NASC monomer

The synthesis method of the NASC monomer is the same as that previously reported[1, 2]. Dissolve aminourea hydrochloride (31.75 g) in 30 mL of deionized (DI) water, followed by 168 mL of cold K_2CO_3 solution (2 M) and 90 mL of cold ether in a 500 mL round-bottom flask. Subsequently, 28.50 g of acryloyl chloride in 120 mL of ether was added dropwise to the mixture with stirring at 0°C for about 4 hours. The resulting white precipitate is filtered and washed with cold water to obtain a crude product. The crude product was then dissolved in DI water and stirred at 95 °C for 1 h. After that, the undissolved pellet is removed by centrifugation and the supernatant is lyophilized to obtain the final NASC monomer.

Preparation of PNAGA hydrogel

25 wt% of NAGA monomer was dissolved in deionized water, and Lithium Phenyl(2,4,6-trimethylbenzoyl) phosphinate (LAP, 0.5 wt% of the total mass of the monomer) was added as a photoinitiator. The mixture was irradiated under ultraviolet light for 2 minutes until completely cured to obtain PNAGA hydrogel.

Quenching process of the PNAGA hydrogel

Hydrogel samples are clamped in a custom-made stretching unit equipped with a servo-motor drive system and pre-stretched in deionized water at 50 °C. The strain ratio (6 times the initial length) is systematically programmed via a digital control panel. Immediately after the hydrogel reaches the target elongation, it is quenched in deionized water at 0 °C for 2 minutes to reconstruct the fixed-oriented structure using fast hydrogen bonding. Finally, the quenched hydrogel was transferred to deionized water at 25 °C for equilibration for 7 days to obtain the quenched PNAGA hydrogel.

Supporting Discussion

Chemical structure characterization

Fourier-transform infrared spectroscopy (TFS-66 V/S, Bruker, Germany) of the NASC monomer precursor (**Figure S1**) revealed diagnostic absorption bands at: 3439 (NH), 3332 (NH), 3219 (NH), 3062 (NH), 1683 (C=O), 1623 (C=C), 1594 (NH); **Figure S2 and Figure S3** present the NMR spectra of the NASC monomer. The corresponding positions have been clearly marked in the figures. In the ^{1}H NMR spectrum, the chemical shifts are observed at δ = 9.8 (H_a), 7.9 (H_b), 6.2 (H_c), 6.0 (H_d), and 5.7 (H_e). Similarly, in the ^{13}C NMR spectrum, the signals appear at δ = 164.7 (C_a, -CO-), 159.0 (C_b, -CO-), 130.2 (CH_2-CH-), and 126.6 (CH_2-CH-). These results confirm the successful synthesis of the NASC monomer.

Performance analysis of PNA hydrogels with different monomer contents.

The mechanical analysis of PNA hydrogels with different monomer concentrations revealed a clear trend: as the monomer content increased, the tensile strength at break gradually improved, rising from 3.9 ± 0.4 MPa for PNA_{20} to 9.5 ± 0.4 MPa for PNA_{40} (**Figure S4-S5**). Similarly, the Young's modulus increased from 2.5 ± 0.2 MPa to 5.9 ± 0.2 MPa (**Figure S6**). Notably, the elongation at break first increased and then decreased with higher monomer content, reaching a maximum of 981.4 ± 61.8 % for PNA_{25}. Considering the balance between elongation and strength, PNA_{25} was selected for quenching due to its optimal mechanical properties. This hydrogel exhibited the highest toughness, reaching 32.6 ± 5.4 MJ/m^3 (**Figure S7**).

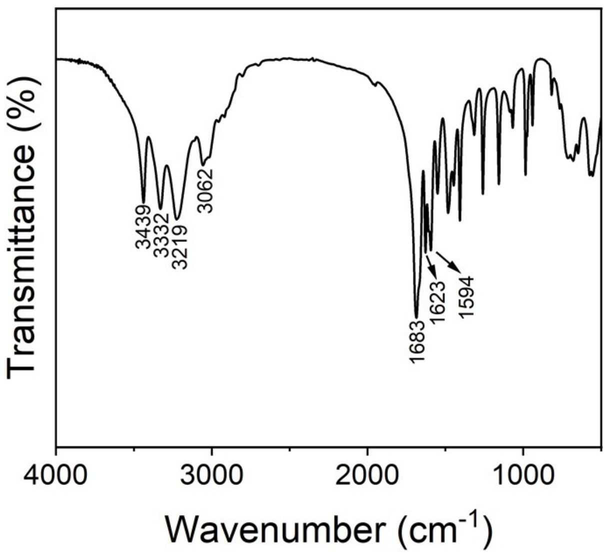

Figure S1. FTIR spectra of the NASC monomer.

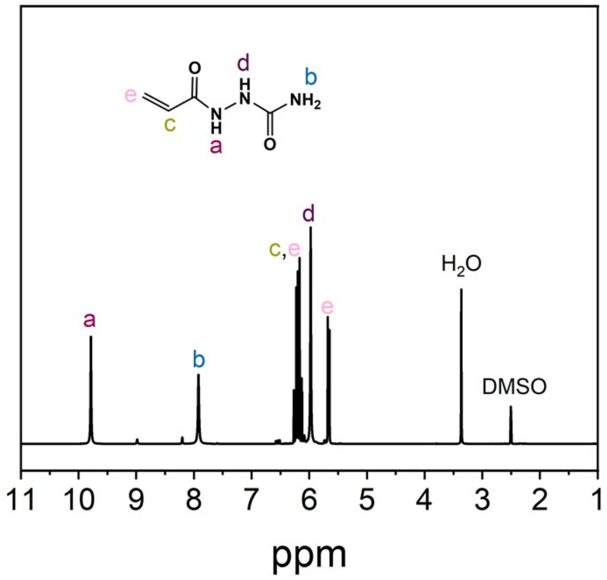

Figure S2. ^{1}H NMR spectra of the NASC monomer.

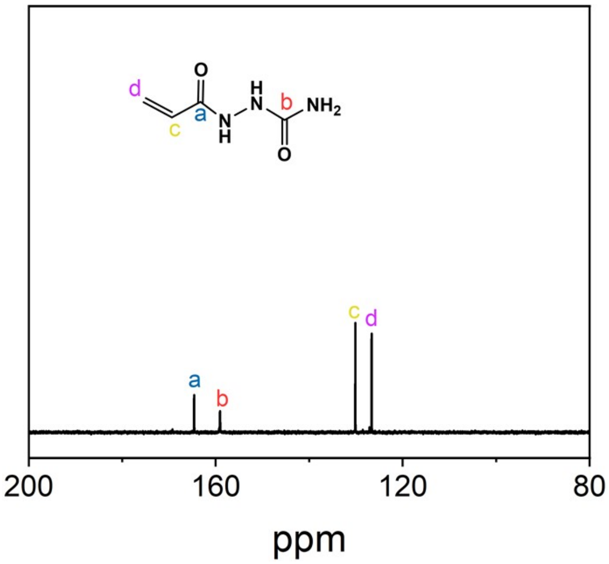

Figure S3. ^{13}C NMR spectra of NASC monomer.

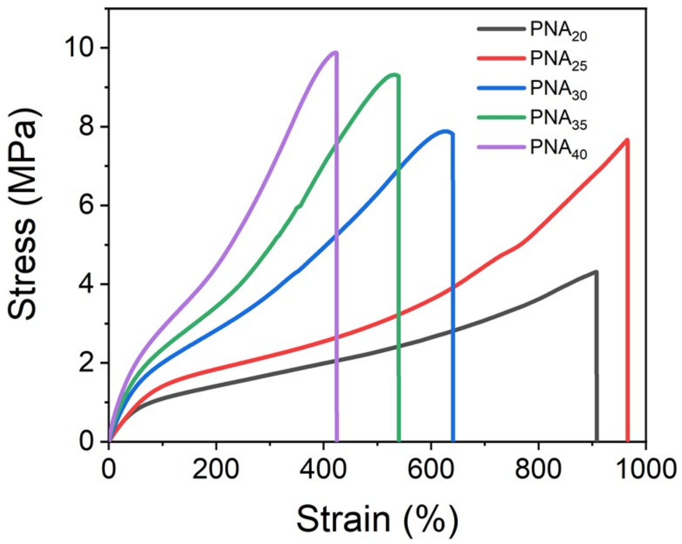

Figure S4. Stress-strain curves of PNA hydrogels with different monomer contents.

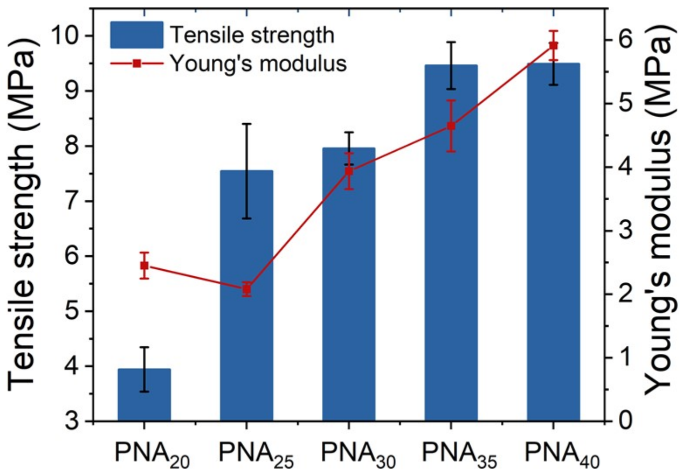

Figure S5. Tensile strength and Young's modulus of PNA hydrogels with different monomer contents. Error bars represent the standard deviation (n = 3). The data is presented as mean values ± SD.

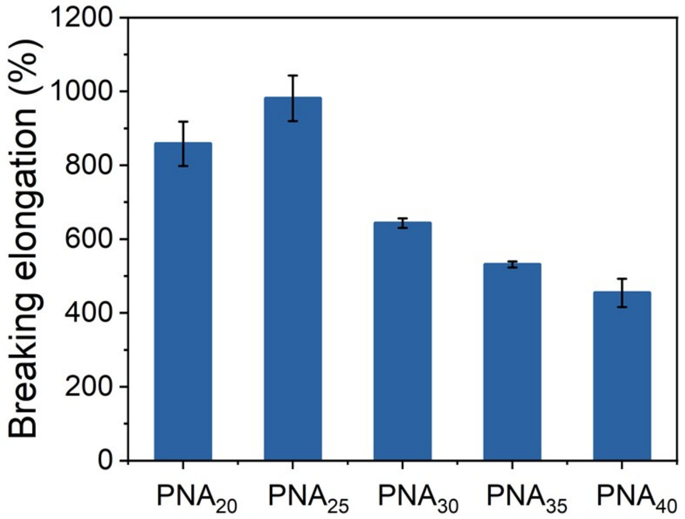

Figure S6. Tensile elongation at break of PNA hydrogels with different monomer contents. Error bars represent the standard deviation (n = 3). The data is presented as mean values ± SD.

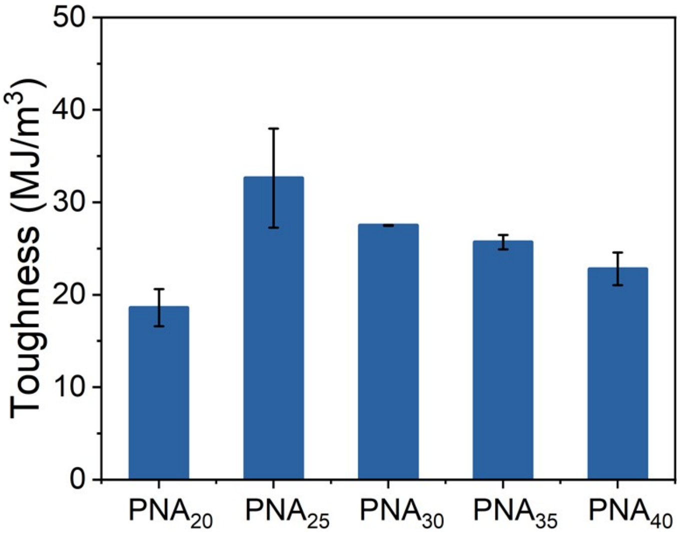

Figure S7. Toughness of PNA hydrogels with different monomer contents. Error bars represent the standard deviation (n = 3). The data is presented as mean values ± SD.

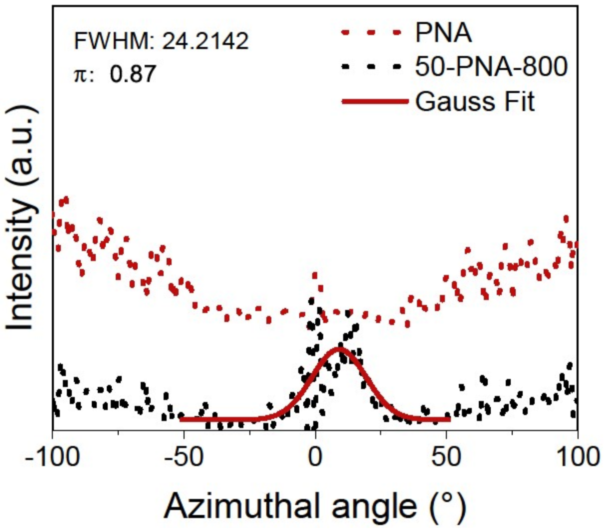

Figure S8. 2D SAXS patterns' corresponding azimuthal angle plots of the hydrogel.

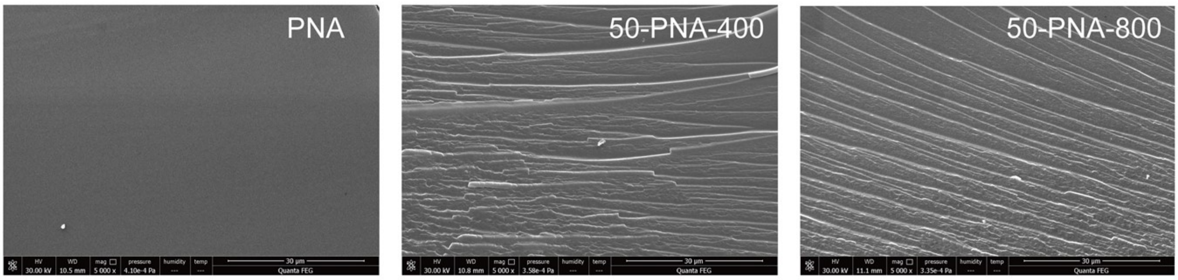

Figure S9. The SEM image of PNA, 50-PNA-400 and 50-PNA-800.

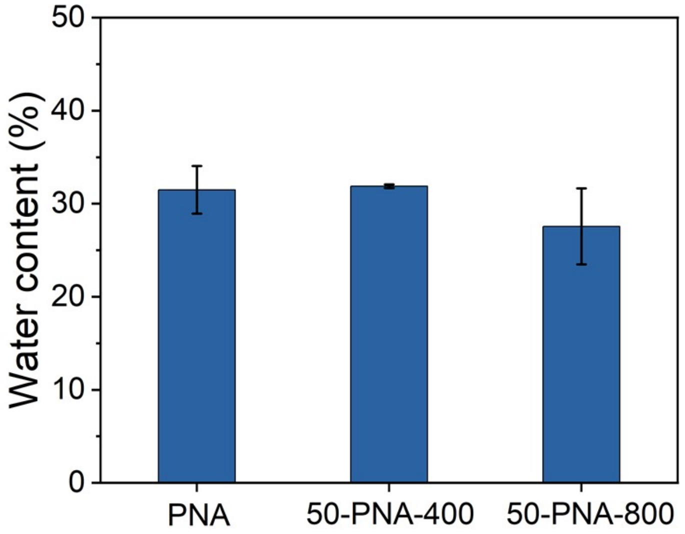

Figure S10. The water content of the pristine PNA, 50-PNA-400 and 50-PNA-800. Error bars represent the standard deviation (n = 3). The data is presented as mean values ± SD.

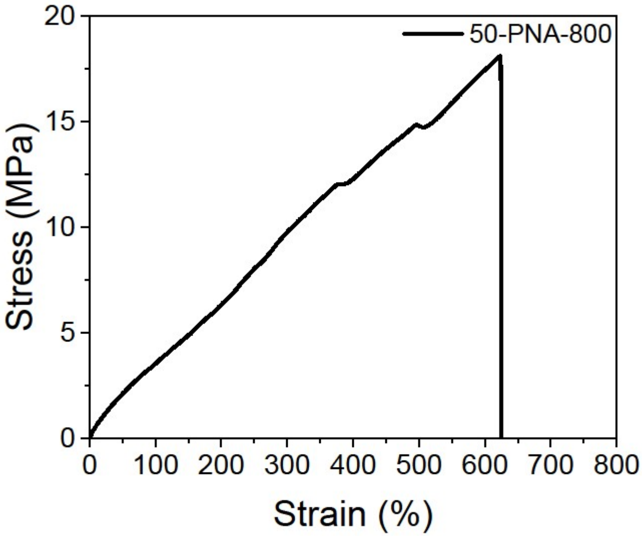

Figure S11. Stress-strain curves of 50-PNA-800 hydrogels after 14 days of immersion in water post-quenching.

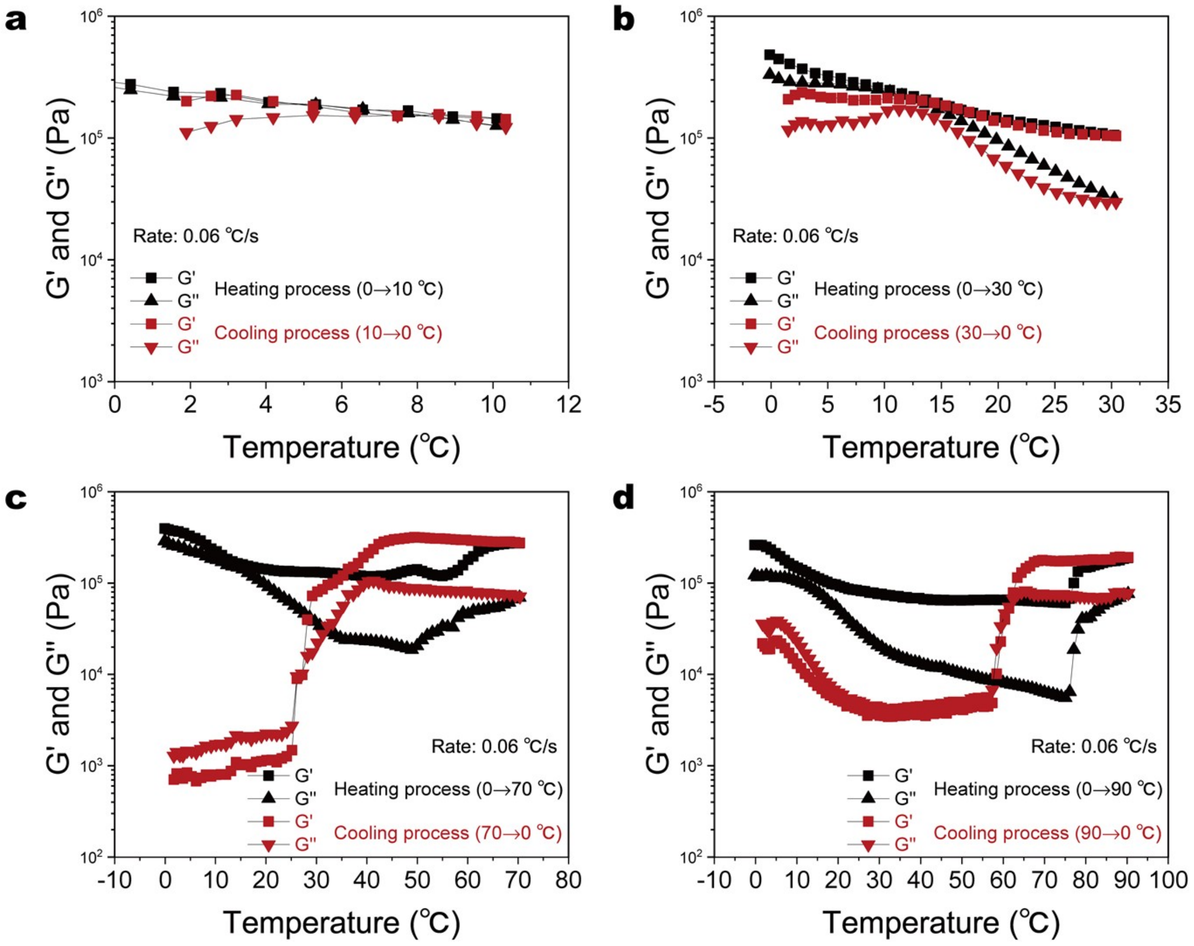

Figure S12. (a) Rheological test of PNA hydrogel from 0 °C to 10 °C and back from 10 °C to 0 °C. (b) Rheological test of PNA hydrogel from 0 °C to 30 °C and back from 30 °C to 0 °C. (c) Rheological test of PNA hydrogel from 0 °C to 70 °C and back from 70 °C to 0 °C. (d) Rheological test of PNA hydrogel from 0 °C to 90 °C and back from 90 °C to 0 °C. The frequency of 1 Hz and a strain is 1%.

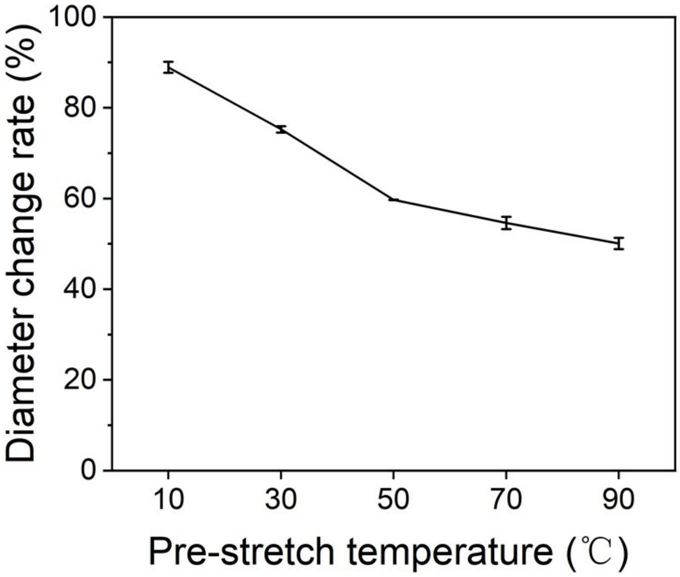

Figure S13. The diameter changes rate of hydrogels quenched after being stretched to 800% at different temperatures at 7 days. Error bars represent the standard deviation (n = 3). The data is presented as mean values ± SD.

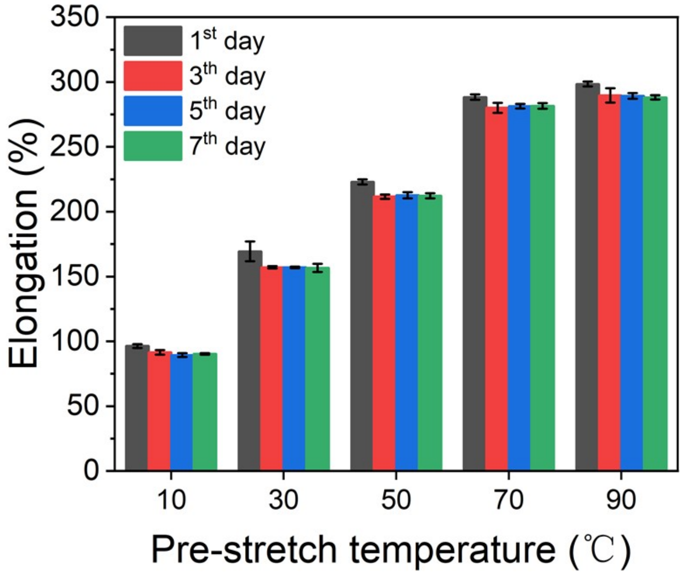

Figure S14. The elongation of hydrogels quenched after being stretched to 800% at different temperatures at 1 day to 7 days. Error bars represent the standard deviation (n = 3). The data is presented as mean values ± SD.

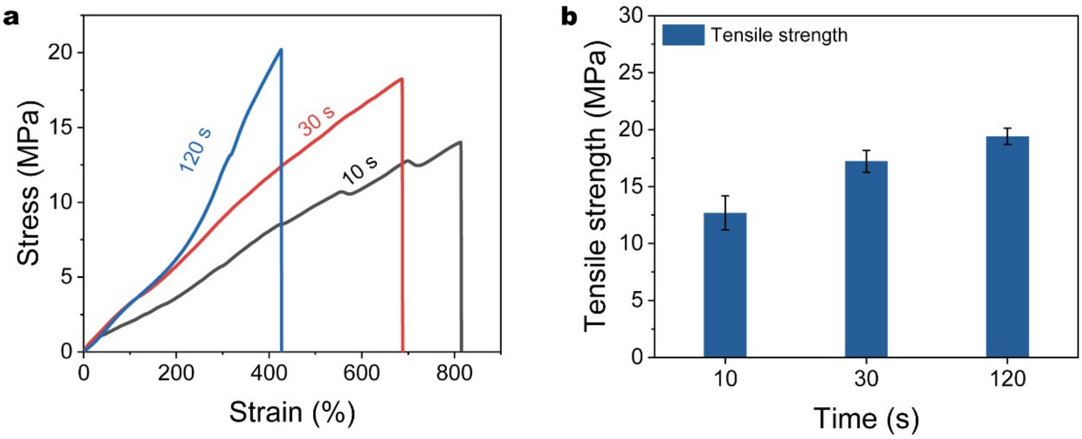

Figure S15. (a) Stress-strain curves and corresponding (b) tensile strength of 50-PNA-800 hydrogels quenched in 0 °C water under different time. Error bars represent the standard deviation (n = 3). The data is presented as mean values ± SD.

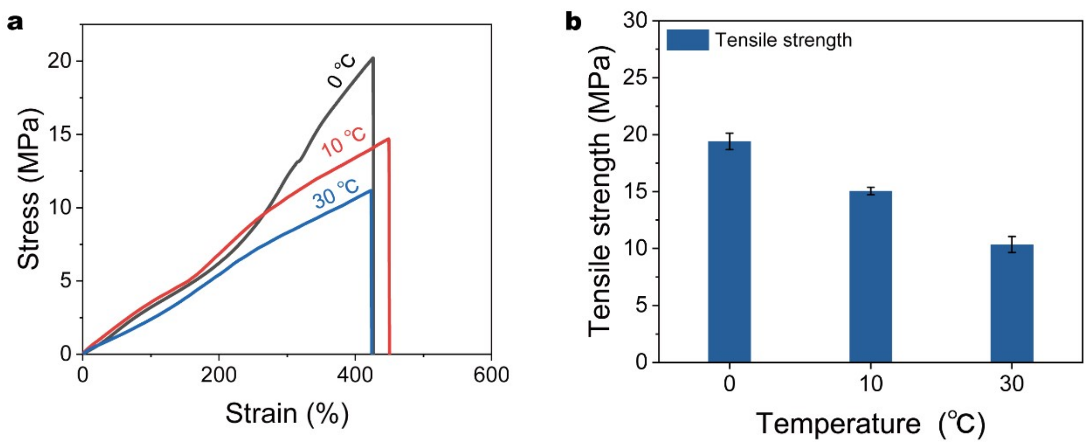

Figure S16. (a) Stress-strain curves and corresponding (b) tensile strength of 50-PNA-800 hydrogels quenched in water at different temperatures for 2 minutes. Error bars represent the standard deviation (n = 3). The data is presented as mean values ± SD.

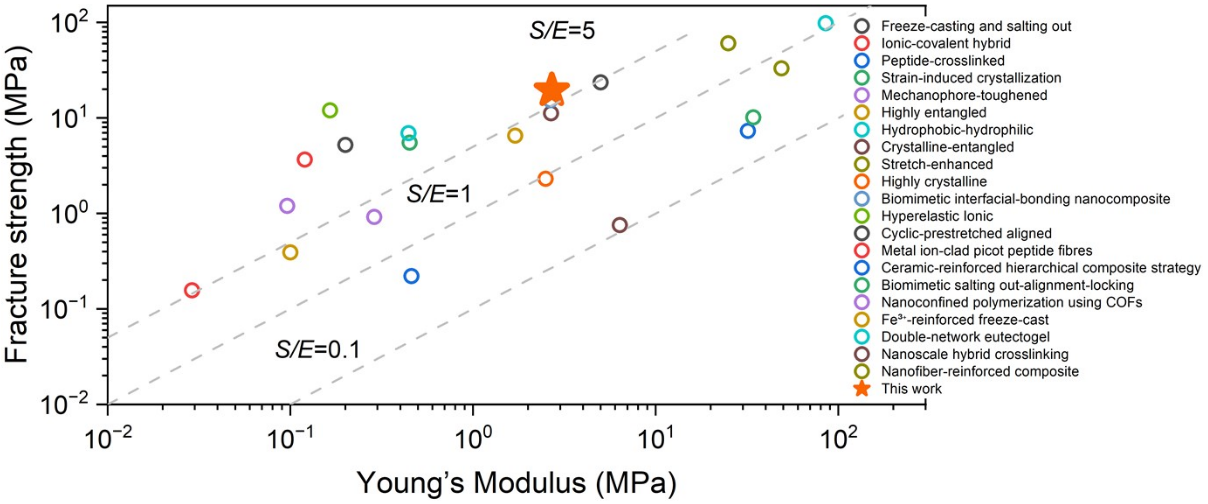

Figure S17. Comparison chart in the Ashby plot of Young's modulus versus fracture strength of quenched hydrogel with other reported strong and tough hydrogel. These hydrogels types include freeze-casting and salting out,[3] ionic-covalent hybrid,[4] peptide-crosslinked,[5] strain-induced crystallization,[6] mechanophore-toughened,[7] highly entangled,[8] hydrophobic-hydrophilic,[9] crystalline-entangled,[10] stretch-enhanced,[11] highly crystalline,[12] biomimetic interfacial-bonding nanocomposite,[13] hyperelastic ionic[14] cyclic-prestretched aligned,[15] metal ion-clad picot peptide fibres,[16] ceramic-reinforced hierarchical composite strategy,[17] biomimetic salting out-alignment-locking,[18] nanoconfined polymerization using COFs,[19] Fe^{3+}-reinforced freeze-cast,[20] double-network eutectogel,[21] nanoscale hybrid crosslinking,[22] nanofiber-reinforced composite.[23]

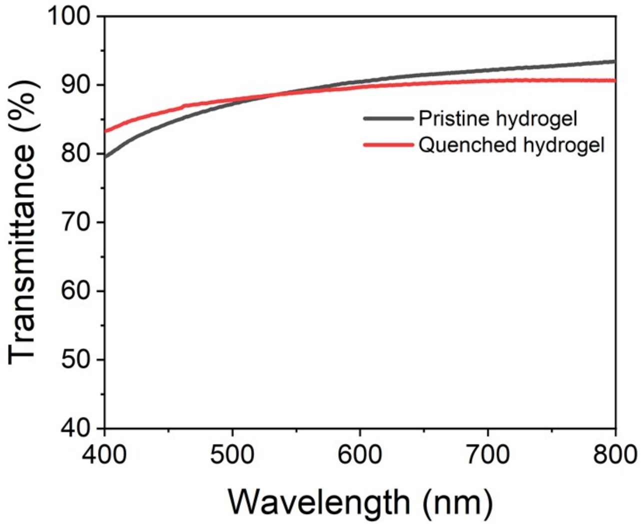

Figure S18. The transmittance of the pristine and quenched hydrogel (50-PNA-800).

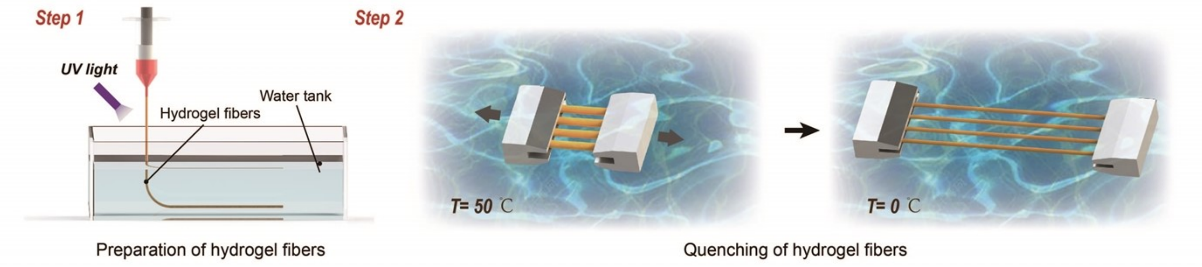

Figure S19. Schematic diagram of hydrogel fiber extrusion, pre-stretching and quenching process.

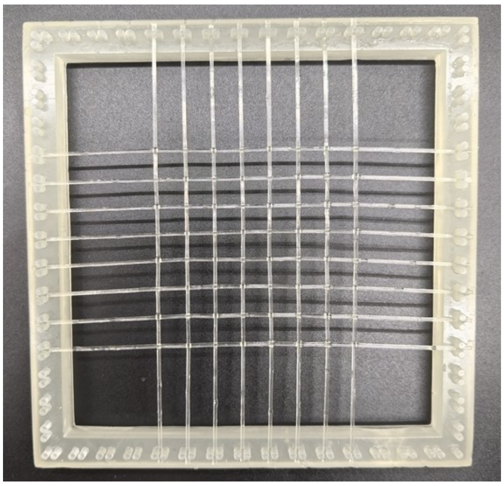

Figure S20. Drop-weight impact test of the hydrogel fiber mesh. The dimensions of the outer frame are 10 cm × 10 cm.

References

1. C. Fan, B. Liu, Z. Xu, C. Cui, T. Wu, Y. Yang, D. Zhang, M. Xiao, Z. Zhang and W. Liu, *Mate. Horiz.*, 2020, **7**, 1160-1170.
2. J. Wu, Y. Wang, P. Jiang, X. Wang, X. Jia and F. Zhou, *Nat. Commun.*, 2024, **15**, 3482.
3. M. Hua, S. Wu, Y. Ma, Y. Zhao, Z. Chen, I. Frenkel, J. Strzalka, H. Zhou, X. Zhu and X. He, *Nature*, 2021, **590**, 594-599.
4. J. Y. Sun, X. Zhao, W. R. Illeperuma, O. Chaudhuri, K. H. Oh, D. J. Mooney, J. J. Vlassak and Z. Suo, *Nature*, 2012, **489**, 133-136.
5. P. Liu, Y. Zhang, Y. Guan and Y. Zhang, *Adv. Mater.*, 2023, **35**, e2210021.
6. C. Liu, N. Morimoto, L. Jiang, S. Kawahara, T. Noritomi, H. Yokoyama, K. Mayumi and K. Ito, *Science*, 2021, **372**, 1078-1081.
7. Z. Wang, X. J. Zheng, T. Ouchi, T. B. Kouznetsova, H. K. Beech, S. Av-Ron, T. Matsuda, B. H. Bowser, S. Wang, J. A. Johnson, J. A. Kalow, B. D. Olsen, J. P. Gong, M. Rubinstein and S. L. Craig, *Science*, 2021, **374**, 193-196.
8. J. Kim, G. G. Zhang, M. X. Shi and Z. G. Suo, *Science*, 2021, **374**, 212-+.
9. G. G. Zhang, J. Steck, J. Kim, C. H. Ahn and Z. G. Suo, *Sci. Adv.*, 2023, **9**.
10. Y. Wu, Y. Zhang, H. Wu, J. Wen, S. Zhang, W. Xing, H. Zhang, H. Xue, J. Gao and Y. Mai, *Adv. Mater.*, 2023, **35**, e2210624.
11. X. Wang, S. Zheng, J. Xiong, Z. Liu, Q. Li, W. Li and F. Yan, *Adv. Mater.*, 2024, **36**, e2313845.
12. S. T. Lin, X. Y. Liu, J. Liu, H. Yuk, H. C. Loh, G. A. Parada, C. Settens, J. Song, A. Masic, G. H. McKinley and X. H. Zhao, *Sci. Adv.*, 2019, **5**.
13. B. Bao, Q. Zeng, K. Li, J. Wen, Y. Zhang, Y. Zheng, R. Zhou, C. Shi, T. Chen, C. Xiao, B. Chen, T. Wang, K. Yu, Y. Sun, Q. Lin, Y. He, S. Tu and L. Zhu, *Nat. Mater.*, 2023, **22**, 1253-1260.
14. L. L. Chen, Z. K. Jin, W. W. Feng, L. Sun, H. Xu and C. Wang, *Science*, 2024, **383**, 1455-1461.
15. S. Lin, J. Liu, X. Liu and X. Zhao, *Proc. Natl. Acad. Sci. U.S.A.*, 2019, **116**, 10244-10249.
16. B. Xue, Z. Bashir, Y. Guo, W. Yu, W. Sun, Y. Li, Y. Zhang, M. Qin, W. Wang and Y. Cao, *Nat. Commun.*, 2023, **14**, 2583.
17. Q. Liu, X. Dong, H. Qi, H. Zhang, T. Li, Y. Zhao, G. Li and W. Zhai, *Nat. Commun.*, 2024, **15**, 3237.
18. X. Sun, Y. Mao, Z. Yu, P. Yang and F. Jiang, *Adv. Mater.*, 2024, **36**, e2400084.
19. W. Li, X. Wang, Z. Liu, X. Zou, Z. Shen, D. Liu, L. Li, Y. Guo and F. Yan, *Nat. Mater.*, 2024, **23**, 131-138.
20. X. Guo, X. Y. Dong, G. J. Zou, H. J. Gao and W. Zhai, *Sci. Adv.*, 2023, **9**.
21. C. Xu, A. Xie, H. Hu, Z. Wang, Y. Feng, D. Wang and W. Liu, *Nat. Commun.*, 2025, **16**, 2589.
22. S. Lin, C. Cao, Q. Wang, M. Gonzalez, J. E. Dolbow and X. Zhao, *Soft Matter*, 2014, **10**, 7519-7527.
23. H. Zhuo, X. Dong, Q. Liu, L. Hong, Z. Zhang, S. Long and W. Zhai, *Nat. Commun.*, 2025, **16**, 980.

Video S1. Comparative tensile behavior of the pristine versus unquenched hydrogels

Video S2. Drop-weight impact test of the hydrogel fiber meshs
